# Protein Repair and Degradation during Aging

**DOI:** 10.1100/tsw.2002.98

**Published:** 2002-01-29

**Authors:** Bertrand Friguet

**Affiliations:** Laboratoire de Biologie et Biochimie Cellulaire du Vieillissement, Université Denis Diderot-Paris 7, CC 7128, 2 Place Jussieu, 75251 Paris Cedex 05, France

**Keywords:** aging, protein maintenance, protein oxidative damage, protein degradation, protein repair, proteasome, peptide methionine sulfoxide reductase

## Abstract

Cellular aging is characterized by a build-up of oxidatively modified proteins. The steady-state level of oxidized proteins depends on the balance between the rate of protein oxidative damage and the rates of protein degradation and repair. Therefore, the accumulation of oxidized protein with age can be due to increased protein damage, decreased oxidized protein degradation and repair, or the combination of both mechanisms. The proteasomal system is the major intracellular proteolytic pathway implicated in the degradation of oxidized protein, and the peptide methionine sulfoxide reductase catalyzes the reduction of methionine sulfoxide (i.e., oxidized methionine) to methionine within proteins. A short summary on protein oxidative damage and oxidized protein degradation is given, and evidence for a decline of proteasome function with age is presented. Arguments for the implication of peptide methionine sulfoxide reductase in the age-related accumulation of oxidized protein are also discussed.

## INTRODUCTION

The aging process is characterized by an increased accumulation of damaged macromolecules, and oxidized protein build-up is considered to be a hallmark of cellular aging[1,2]. The age-related accumulation of altered protein raises the problem of the efficacy of intracellular protein maintenance, in particular the protein degradation and the protein repair systems. Indeed, these systems that take care of the removal or repair of damaged proteins appear to be affected with aging and therefore to directly contribute to the increased intracellular load of functionally impaired protein that will ultimately lead to a global deterioration of the cellular machinery. Since cytosolic oxidized protein degradation and basal protein turnover have been shown to be mostly carried out by the proteasomal system[3,4,5], the fate of proteasome in aging is mainly addressed in this mini-review. In addition, the role of the peptide methionine sulfoxide reductase, one of the very few protein-repair enzymes described that has recently received attention[6,7], and its possible implication in the age-related decline of protein maintenance is also discussed.

## PROTEIN DAMAGE AND AGING

Damage to macromolecules occurring upon aging are mainly produced through reactions implicating reactive oxygen species such as the superoxide anion ($O_2^{\cdot -}$), hydrogen peroxide ($H_2O_2$), and the hydroxyl radical ($OH^{\cdot}$)[1]. These reactive species (also referred to as free radicals) are produced at different levels inside the cell and are generated through the metabolism of cellular organelles, such as the mitochondria and the peroxisomes. In addition, certain extrinsic factors, such as UV irradiation or toxins, can participate in the increased production of intracellular free radicals. Antioxidant systems, both enzymatic and nonenzymatic, contribute to the regulation of the level of reactive oxygen species by trapping these free radicals and maintaining the cellular homeostasis. When the equilibrium between production and elimination of free radicals is disrupted, the increased concentration of these deleterious factors will damage all macromolecules (nuclear and mitochondrial DNA, proteins, and lipids), leading to irreversible modifications. Concerning proteins, almost all amino acids can be oxidized by reactive oxygen species, sulfur-containing (cysteine and methionine) and aromatic (tyrosine and tryptophan) amino acids being the most susceptible[8,9,10]. Oxidation products of cystein are dissulfide bridges and mixed dissulfides, while methionine is converted to methionine sulfoxide. Oxidation of other amino acids mostly generates hydroxyl and carbonyl groups, and detection of protein-associated carbonyls is a common method for a general assessment of protein oxidation[11,12]. Protein-associated carbonyls are readily derivatized by treatment of protein samples with 2,4-dinitrophenyl hydrazine (DNPH). Oxidized proteins are generally less active and less thermostable than their native forms. Oxidized proteins have also been shown to expose hydrophobic residues at the surface of the protein, as revealed by increased binding of the fluorescent probe 8-anilino-1-naphthalene sulfonic acid[13], and to exhibit a decreased thermodynamic stability[14]. Other mechanisms, such as glycation, glycoxidation, and conjugation with lipid peroxidation products are also contributing to the alteration of macromolecules, mostly proteins. These modifications, which also introduce carbonyl groups in proteins, are resulting from the covalent binding of sugars and their oxidation products as well as aldehydes originating from oxidation of membrane polyunsaturated fatty acids oxidation[15]. Reaction of proteins with these compounds, as well as highly oxidized proteins, can lead to the formation of intra- and/or intermolecular cross-linking. Using the carbonyl content as an index for the protein oxidation status, a significant increase of oxidized protein with age has been reported in human dermal fibroblasts, human keratinocytes, human erythrocytes, human brain, rat hepatocytes, as well as in *Drosophila*[16,17,18,19,20]. This age-related accumulation of damaged protein, in association with the impairment of important enzymes, is therefore believed to affect cellular integrity. For instance, cataract formation results, at least partly, from the accumulation of oxidized proteins in the lens[21], and the reversal of the age-related loss of spatial memory by chronic injection of the free radical scavenger tert-butyl-α-phenylnitrone in gerbils has been attributed to the treatment-induced reduction of oxidized proteins in the brain[22]. Oxidatively damaged protein accumulation has also been associated with age-related diseases such as Parkinson and Alzheimer’s diseases, lateral amyotrophic sclerosis, and rheumatoid arthritis[2].

## OXIDIZED PROTEIN DEGRADATION AND OXIDATIVE STRESS

Oxidatively modified proteins are preferentially degraded *in vitro* by the 20S proteasome in an ATP-independent fashion[23,24], while covalent attachment of ubiquitin marks protein for ATP-dependent degradation by the 26S proteasome[25,26]. The proteasome is a high-molecular weight multienzymatic proteolytic complex present in the cytosol and nucleus of eukaryotic cells. It is now well documented that oxidized protein degradation is mainly achieved by the proteasome in an ATP- and ubiquitin-independent manner[4], although other studies have shown that the removal of oxidatively damaged proteins from lens cells is carried out by the ubiquitin-26S proteasome pathway[27,28]. It is also interesting to note that carboxymethyl-lysine modified proteins (formed upon glycoxidation), although resistant to proteolysis by the 20S proteasome *in vitro*, were found to be ubiquitinated in glyoxal-treated human dermal fibroblasts[29]. Protein modified by the lipid peroxidation product 4-hydroxy-2-nonenal (HNE) have also been reported to undergo ubiquitination upon ferric nitriloacetate-induced oxidative stress in the kidney[30]. Both 20S and 26S proteasome cleave substrate proteins at the carboxyl end of hydrophobic (chymotrysin-like activity), basic (trypsin-like activity), and acidic (peptidylglutamyl peptide hydrolase activity) amino acids. The 20S proteasome is the catalytic core of the 26S proteasome and is made of 2 x 14 subunits arranged as four stacked rings. The outer rings are formed by noncatalytic $\alpha$ subunits, and the two inner rings are formed by catalytic $\beta$ subunits. The 26S proteasome is formed upon association of the 20S proteasome with PA 700 (19S regulatory complex)[3,5]. The proteasome has been implicated in a variety of processes, including protein turnover and oxidized protein degradation, but also processing of antigens, regulation of stress response pathways, cell differentiation, proliferation, and apoptosis[31,32,33,34,35,36,37,38]. Upon metal-catalyzed oxidation *in vitro*, both trypsin-like and peptidylglutamyl peptide hydrolase activity of the 20S proteasome are readily inactivated[39]. This inactivation is dependent on whether the 20S proteasome is in its latent or active form before exposure to the reactive oxygen species-generating system[40]. Indeed, when the proteasome is in its latent form, treatment with metal-catalyzed oxidation resulted in activation of proteasome peptidase activities. This finding may be related to the differential susceptibility of the 26S proteasome vs. 20S proteasome oxidative inactivation, the 26S proteasome being more sensitive to oxidative inactivation than the 20S proteasome[41]. Nitric oxide has also been shown to inactivate the chymotrypsin-like activity for the 26S proteasome[42]. Selective inactivation of the trypsin-like peptidase activity was observed upon treatment with the lipid peroxidation HNE *in vitro*[43]. HNE-mediated impairment of proteasome function has recently been shown *in vivo* since oxidative stress in kidney[30] and ischemia-reperfusion in brain[44] were correlated with increased HNE covalent binding to the proteasome. Moreover, specific modification by HNE of three proteasome subunits has been associated with inactivation of the proteasome trypsin-like peptidase activity upon coronary occlusion-reperfusion[45]. In addition, treatment of the model protein glucose-6-phosphate dehydrogenase with HNE results in the formation of intramolecular cross-linking and in its resistance to proteolysis by the 20S proteasome together with the ability of the modified protein to inhibit the 20S proteasome in a noncompetitive fashion[46,47]. Proteasome inhibition upon cell loading with artificial lipofuscin or ceroid pigment argues for a direct implication of such oxidized cross-linked protein in senescence-associated impairment of proteasome function[48].

## PROTEASOME AND AGING

The age-related slowing down of protein turnover and the accumulation of oxidized proteins raise the possibility that the proteasome degradation pathway is impaired with age (for recent reviews see[49,50,51,52]). Indeed, we and others have reported that proteasome activity is declining with age in a variety of tissues[17,39,53,54,55], while other studies have shown that impairment of proteasome function may not be universal[54,56]. In addition, events leading to the loss of proteasome function have not been yet fully characterized. Concerning the ubiquitination system, the activities of the ubiquitinating enzymes E1, E2, and E3 do not show any consistent change with age, while accumulation of high molecular weight protein-ubiquitin conjugates is generally associated with aging and certain age-related diseases[25]. Therefore, the observed accumulation of protein-ubiquitin conjugates most likely reflects a defect in the proteasomal system. Evidence has been provided that both 20S and 26S proteasome peptidase activities are affected by age[50].

We have previously reported that the peptidylglutamyl peptide hydrolase activity is decreased for 20S proteasome purified from the liver of old Fisher 344 and LOU rats indicating that direct inactivation of a peptidase activity may account for the decreased efficacy of the proteasomal system[39,55]. In recent studies with human epidermal cells and rat myocardiac cells, we have observed that the accumulation of oxidatively modified proteins is associated with a decreased proteasome activity and content, suggesting that proteasome expression is down-regulated with age[17,57]. Further studies on purified 20S proteasome from both tissues have also shown an age-related inactivation of some peptidase activities[57,58]. Age-related variations of gene expression patterns have also been recently reported for both mitotic and postmitotic cells using the microarray technology[59,60]. Less than 2% of the 6347 genes monitored were affected with age under either conditions. Interestingly, in both situations transcription of several genes encoding either 20S or 26S proteasome subunits were found to decline with age. A decline of proteasome activity and content has been reported in human embryonic MRC5 fibroblasts undergoing replicative senescence[61] as well as in fibroblast cultures originated from donors of different ages[62]. In the latter study, healthy centenarians fibroblast cultures were also examined, and they exhibited proteasome subunits expression levels and activity close to those found in young donors cultures, favoring the hypothesis that the sustained activity of the proteasome level in these individuals might have contributed to their longevity. Finally, depending on the cellular system investigated, loss in proteasome activity during aging appears to be due to the combined effects of at least the following: (1) decreased proteasome expression and content, (2) inactivation upon modification of proteasome subunits, and (3) formation of inhibitory proteins. The last point is supported by studies showing that proteasome inhibition is induced upon cell loading with artificial lipofuscin[48] and that the age-dependent decline of certain peptidase activities observed in cytosolic extracts is partially relieved upon purification of the 20S proteasome[57].

## METHIONINE OXIDATION AND REPAIR

Very few enzymatic repair systems for oxidatively damaged proteins have been described. Only sulfur-containing amino acid oxidation products can be reversed within proteins either by the thioredoxin/thioredoxin reductase system for dissulfide bridges and mixed dissulfides reduction[63] or by the peptide methionine sulfoxide reductase (MsrA) for methionine sulfoxide reduction[7,64,65]. It is interesting that those amino acids that are the most susceptible to oxidation can be reversed back to their reduced forms by enzymatic systems that protect the cell against oxidative damage. This protection may either be a directed one allowing repair of important cystein and methionine residues in proteins or, as previously proposed, be part of the reactive oxygen species scavenging systems, wherein proteins would have in this respect a more general antioxidant role[66]. We have recently shown that peptide methionine sulfoxide reductase expression and activity are actually decreased with age in rat organs such as brain, liver, and kidney[67], and peptide methionine sulfoxide reductase is also impaired in Alzheimer's disease[68]. In addition, Moskovitz et al.[69] have just reported that comparison of a mouse strain lacking the MsrA gene with the wild type indicates that peptide methionine sulfoxide reductase is a regulator of antioxidant defense and lifespan in mammals. Taken together, these findings argue for an important role of this protein repair enzyme in the aging process and in the age-related decline of protein maintenance.

In conclusion, both proteasome and peptide methionine sulfoxide reductase functions appear to be impaired with age and in certain age related diseases. Therefore, the next goal will be to more precisely define the relevant mechanisms implicated in this protein maintenance age-related decline in order to propose strategies aimed at preserving this crucial function.

## ACKNOWLEDGMENTS

The work from our laboratory is supported by funds from MENRT (Institut Universitaire de France and Université Denis Diderot-Paris7), the Fondation pour la Recherche Médicale and a European 5th Framework Program Grant (QLK6-CT1999-02193).
